# Beyond patient perception: PROMs correlate with objective functional tests following knee cartilage repair

**DOI:** 10.1186/s12891-026-10123-5

**Published:** 2026-07-13

**Authors:** Niklas Wegerich, Tizian Heinz, Sebastian Frischholz, Annette Eidmann, Ioannis Stratos, Konstantin Horas, Stephan Reppenhagen, Maximilian Rudert, Manuel Weißenberger

**Affiliations:** 1 Department of Orthopaedic Surgery, Koenig-Ludwig-Haus, University of Wuerzburg, Wuerzburg, 97074 Germany; 2 Frankfurt Centre for Bone Health and Endocrinology, Frankfurt, 60313 Germany

**Keywords:** Knee cartilage repair, Patient-reported outcome measures (PROMs), Functional performance, Hop tests, Limb symmetry index, IKDC, KOOS, Rehabilitation, Return to sports, Fnee function

## Abstract

**Background:**

Patient-reported outcome measures (PROMs) are widely used to evaluate subjective knee function after cartilage repair surgery, but their association with objective functional performance is unclear. This study explored correlations between PROMs and functional performance tests in patients after knee cartilage repair.

**Methods:**

In this monocentric observational study, 52 patients from the German Cartilage Registry underwent standardized functional testing at a mean follow-up of 66.8 months. PROMs included the subjective IKDC score and KOOS subscales. Functional performance comprised isometric strength testing, single-leg hop tests (SLHD), Timed Up and Go (TUG), Timed 6-Meter Hop (T6H), 30-second Sit-to-Stand, and Single Leg Stance. Spearman correlations were calculated between PROMs and limb symmetry indices or performance categories.

**Results:**

Dynamic unilateral hop tests showed the strongest associations with PROMs, with SLHD symmetry correlating strongly with IKDC (ρ = 0.540) and moderately with all KOOS subscales. T6H performance also correlated strongly with IKDC and KOOS Sport. Knee extension strength showed moderate correlations with IKDC and KOOS Sport, whereas flexion strength and low-demand tests (TUG, Single Leg Stance) showed no significant associations.

**Conclusion:**

PROMs demonstrate moderate, test-dependent correlations with objective functional performance after knee cartilage repair, particularly for dynamic unilateral tasks. PROMs reflect functional impairment but cannot replace objective testing. Therefore, a combined assessment approach is recommended.

## Introduction

Focal cartilage defects of the knee are common and frequently result in persistent functional deficits, reduced load tolerance, pain, and limitations in sport and recreational activities [1, 2]. Central aims of cartilage repair surgery therefore include the restoration of functional capacity and the ability to tolerate mechanical load in daily life and sports [3–5]. Outcome evaluation after cartilage repair surgery commonly relies on patient-reported outcome measures (PROMs) and functional performance tests [5–7].

PROMs play a particularly prominent role in clinical research and medical registries as they provide a low-threshold and scalable approach for outcome assessments in large patient cohorts [6, 8–10]. They function as primary endpoint in studies across various medical specialties, including orthopedics [6, 10–13]. These standardized questionnaires aim to capture patients’ subjective perceptions of symptoms, function, and quality of life. However, PROMs may be limited in their ability to reflect objective functional performance capabilities as responses can be influenced by psychological factors, patient expectations, and prior experiences [14–16].

In contrast, objective functional performance tests provide a direct assessment of knee function by quantifying parameters such as strength, explosive power, coordination and tolerance under unilateral conditions [17, 18]. In particular, asymmetric deficits following knee surgery and performance in high-demand unilateral tests such as hop tests are considered clinically relevant [18, 19]. Results in those tests are commonly used to inform clinical decision making in rehabilitation management and return to sports [14, 18]. Therefore, discrepancies in results between PROMs and objective functional performance may lead to over- or underloading in rehabilitation or cause misinterpretation of registry data, when associations are not well understood.

Previous studies have reported inconsistent associations between PROMs and objective data, predominantly focusing on imaging-based scores or clinical-examination findings [7, 15, 20]. While the relationship between PROMs and functional data after anterior cruciate ligament reconstruction has partly been investigated, comparable data for patients after knee cartilage repair surgery remain scarce [14, 21]. In a previous analysis of our cohort, only moderate correlations were observed between PROMs and a clinical score derived from the 2000 IKDC Knee Cartilage Examination form [20].

The research question of the present study was whether PROMs are more closely associated with objective functional performance than with clinical examination findings, which have been addressed previously in this cohort [20]. The aim was to explore the associations between the subjective IKDC score, the KOOS with its five subscales, and a comprehensive set of established functional performance tests. The analysis focused side-to-side asymmetries between the index and contralateral limb and was conducted in an exploratory and hypothesis-generating manner.

## Materials and methods

### Study design and setting

This study is a monocentric observational study with a combined retrospective and prospective study design. The retrospective data were obtained from the German cartilage registry (KnorpelRegister DGOU) and included PROMs, specifically the KOOS score with its subscales, as well as basic patient and defect characteristics. Prospective data were collected during a standardized in-person clinical and functional follow-up examination. All follow-up assessments were carried out at a university orthopedic center by a specifically trained and supervised final-year medical student. This ensured minimal interindividual rater variability. The investigator was not blinded to the patients’ medical history or clinical registry data.

Part of the underlying dataset has been analyzed and reported previously, however the focus of the present study differs substantially [20]. While the prior publication focused on the relationship between PROMs and an objective clinical knee score, the present study addresses a distinct research question by exploring the associations between PROMs and functional performance-based outcome measures.

### Study population

The study population was recruited from the German cartilage registry. This monocentric subcohort included patients who underwent cartilage repair surgery of the knee at the König-Ludwig-Haus, Würzburg, Germany. Surgical procedures comprised arthroscopic debridement, autologous chondrocyte transplantation (ACT), ACT combined with subchondral bone reconstruction, osteochondral transplantation (OCT) and bone marrow stimulation techniques. The distribution of surgical procedures is presented in Table 1. Eligible patients comprised all individuals registered in the knee cartilage registry who had undergone surgical cartilage repair of the knee and agreed to participate in an in-person follow-up examination. Patients were contacted via email or telephone. All patients meeting these criteria and providing complete PROM data for at least one of the analyzed outcome measures were included in this study. Exclusion criteria were limited to decline of participation or unavailability for follow-up. Follow-up examinations were performed at varying time points after surgery and were not scheduled at a standardized postoperative interval.

### Patient-Reported Outcome Measures (PROMs)

The present study is based on frequently used and validated PROMs to assess subjective knee function, namely the subjective IKDC score and the KOOS score. These PROMs serve, among other things, as subjective reference measures for functional performance capability.

The subjective IKDC score is a standardized questionnaire for patient self-evaluation and was obtained on the same day as the clinical and functional follow-up examination. Therefore, it was available for all the patients without temporal discrepancy.

The KOOS consists of the five subscales (Pain, Symptoms, Activities of Daily Living (ADL), Sport and Quality of Life (QoL)). KOOS data were obtained from the German cartilage registry, and for each patient the most recent available score was used to minimize temporal latency to the follow-up examination.

PROMs reflect the patient’s subjective perception of knee function, symptoms and quality of life. In the present study, PROMs were used as a comparative measure in relation to objective, examiner-collected functional performance tests.

### Functional performance assessment

All functional performance tests were conducted according to a standardized protocol, in a fixed order, and on the same day. Unilateral tests were performed for both the index and the contralateral limb. To eliminate confounding factors from varying footwear and ensure standardized conditions, all assessments were performed barefoot. The aim of the assessment was to cover multiple domains of functional knee performance, including strength, explosive power, balance and coordination. No separate physical warm-up protocol was performed. Instead, the fixed testing sequence was designed to provide a progressive active warm-up starting with lower-demand functional tests: (1) isometric strength testing (2), Timed Up and Go Test (TUG) (3), 30-second Sit-to-Stand Test (30s-STS). This was followed by more strenuous unilateral tasks: (4) Single Leg Hop for Distance (SLHD) (5), Timed 6-Meter Hop (T6H), and (6) Timed Single Leg Stance. Rest intervals between individual trials and consecutive tests were self-paced, with the next attempt initiated as soon as the patient communicated subjective readiness.

Isometric strength assessment was carried out to objectively quantify strength differences between index and contralateral knee. Both knee extension and knee flexion strength were assessed. Patients were seated on an examination bench with the knee flexed to 90°. Maximal isometric strength was measured using a hand-held dynamometer (SagaSafe Model AMF-500, accuracy grade 1, Guangzhou Qianqiu Technology Co., CN, Guangzhou, China) and recorded as peak force (N).

Two hop tests were performed: the SLHD and the T6H. For the SLHD, patients were instructed to jump as far as possible on one leg and safely land on the same limb. A trial was considered valid if the landing was stable without additional corrective hops or external support. Two successful attempts per leg were recorded, and the mean jump distance (cm) was used for analysis. The T6H measured the time a patient needed to jump a distance of 6 m on one leg. Two trials per leg were performed, and the mean time (s) was included for analysis.

To assess real life functionality the TUG, 30s-STS and the Timed Single Leg Stance were performed. The TUG measures the time required to rise from a chair, walk three meters, turn, return and sit down again. Patients were instructed to walk at what they considered their normal daily pace. The 30s-STS quantified the number of sit-to-stand repetitions from a chair completed within 30 s without using the arms. For the Timed Single Leg Stance, patients were asked to maintain balance on one leg as long as possible. Two trials per leg were performed, and the mean standing time (s) was documented, with a maximum cut-off of 60 s.

### Objective clinical score

An objective clinical knee score derived from the IKDC 2000 Knee Examination Form was assessed during the same follow-up examination and has been reported previously [20]. As this manuscript focuses exclusively on the association between PROMs and functional performance-based outcome measures, the objective clinical score was not included in the present analysis.

### Statistical analysis

Data analysis and statistical correlations were performed using IBM SPSS Statistics version 29.0.2.0 (IBM Corp., Armonk, NY, USA). Distribution of data was assessed via the Shapiro-Wilk tests and visual inspection of histograms. Metric variables are presented as mean ± standard deviation or median and interquartile range, as appropriate. Categorial variables are reported as absolute numbers or percentages. The significance level was set to α = 0.05.

For single-legged functional tests, limb symmetry indices (LSI) were calculated as quotient of index limb and contralateral limb to quantify functional asymmetries. Correlation analyses were conducted using the Spearman’s rank correlation coefficient due to non-normal data distribution. Effect sizes were interpreted as weak (ρ < 0.3), moderate (0.3 ≤ ρ ≤ 0.5), or strong (ρ > 0.5). Additionally, 95% confidence intervals for the correlation coefficients were calculated via Fisher’s *r*-to-*z* transformation.

The T6H was performed according to the standardized protocol established by Logerstedt et al. [22].The T6H could not be successfully completed by all patients on both limbs. In cases where calculations of side-to-side ratios were not meaningful due to test failure, no artificial numerical values (such as assigning a value of zero) were assigned, as this would imply a perfect performance. Conversely, simply excluding these patients would introduce a substantial selection bias. Instead, patients were categorized into a clinical six-point ordinal scale based on their calculated LSI tiers: Group 1 (LSI < 1.00), Group 2 (LSI 1.00–1.09), Group 3 (LSI 1.10–1.19), Group 4 (LSI 1.20–1.29), Group 5 (LSI > 1.30), and Group 6 (test not possible on the index limb). These categories were treated as ordinal variables coded from 1 to 6 for subsequent statistical analyses, with higher category levels indicating poorer functional performance of the index limb relative to the contralateral side. The categories were defined pragmatically for the present study to ensure a comprehensive representation of our cohort and to systematically include patients who were unable to complete the test.

Missing data were handled by complete-case analyses. Patients with missing values for a specific variable were excluded from the respective analysis but remained included for all other analyses where data were complete. As this study was exploratory in nature, no formal correction for multiple testing was applied and no power calculation was performed beforehand. Consequently, the reported p-values serve as indicators of potential associations rather than confirmatory evidence. Thus, the risk of Type I error inflation must be taken into account.

## Results

### Study population

A total of 52 patients from the German Cartilage Registry were included in the present analysis. The cohort had a mean age of 39.2 years at the time of surgery and was predominantly male. The mean follow-up time was 66.8 months. Detailed demographic and baseline clinical characteristics are summarized in (Table 1).

**Table 1:**
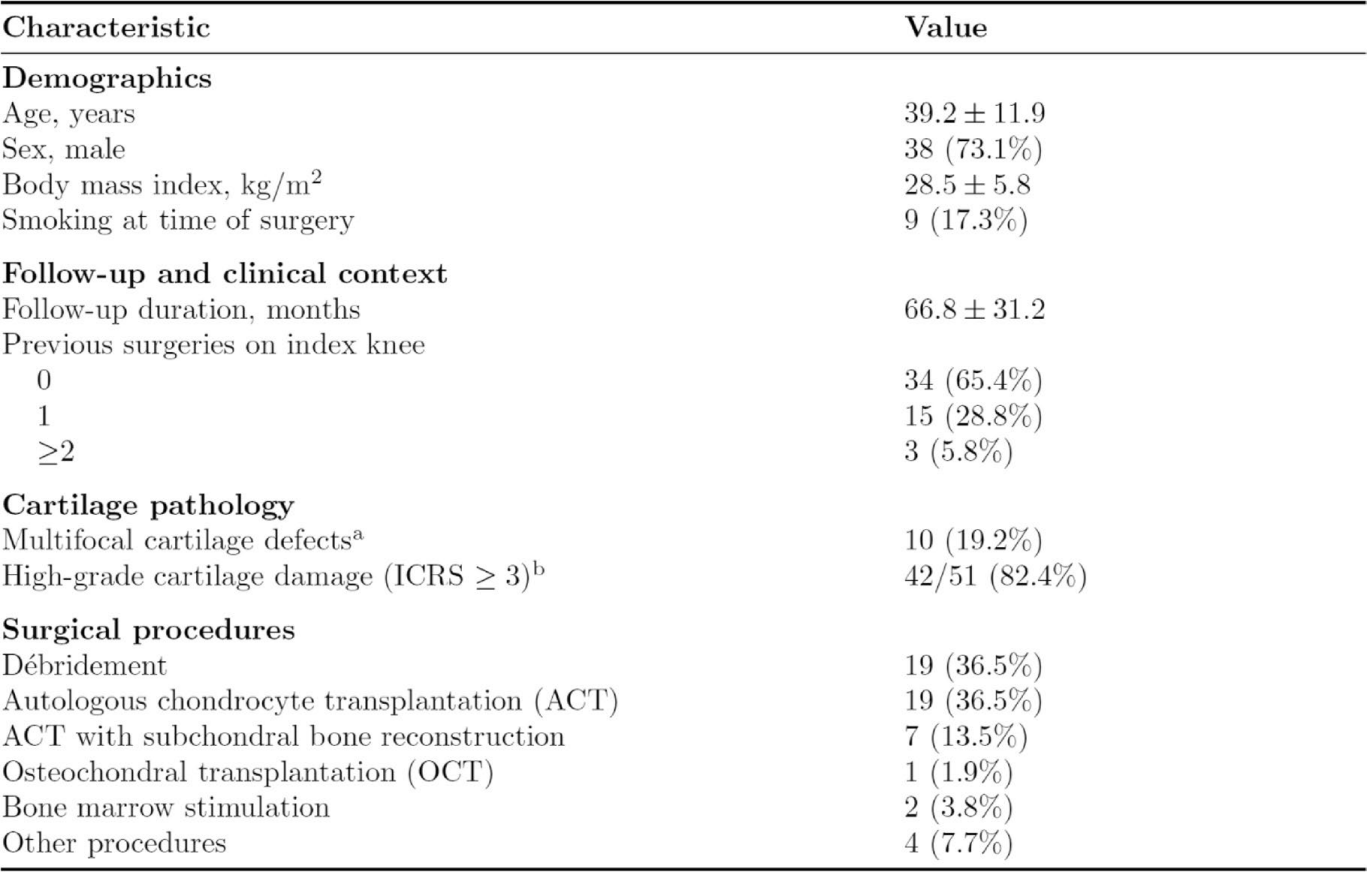
Baseline characteristics of the study cohort (*n* = 52)

Values are presented as mean ± standard deviation or n (%). Smoking status refers to smoking at the time of indexsurgery. Follow-up duration refers to the time interval between index surgery and clinical follow-up

a Multifocal defects defined as ≥2 cartilage defects. b ICRS grade was available for n = 51; high-grade defined as ICRS grade 3-4

*Abbreviations*: *ICRS* International Cartilage Repair Society, *ACT* Autologous Chondrocyte Transplantation, *OCT* Osteochondral Transplantation

### Patient-reported outcome measures

**Fig. 1:**
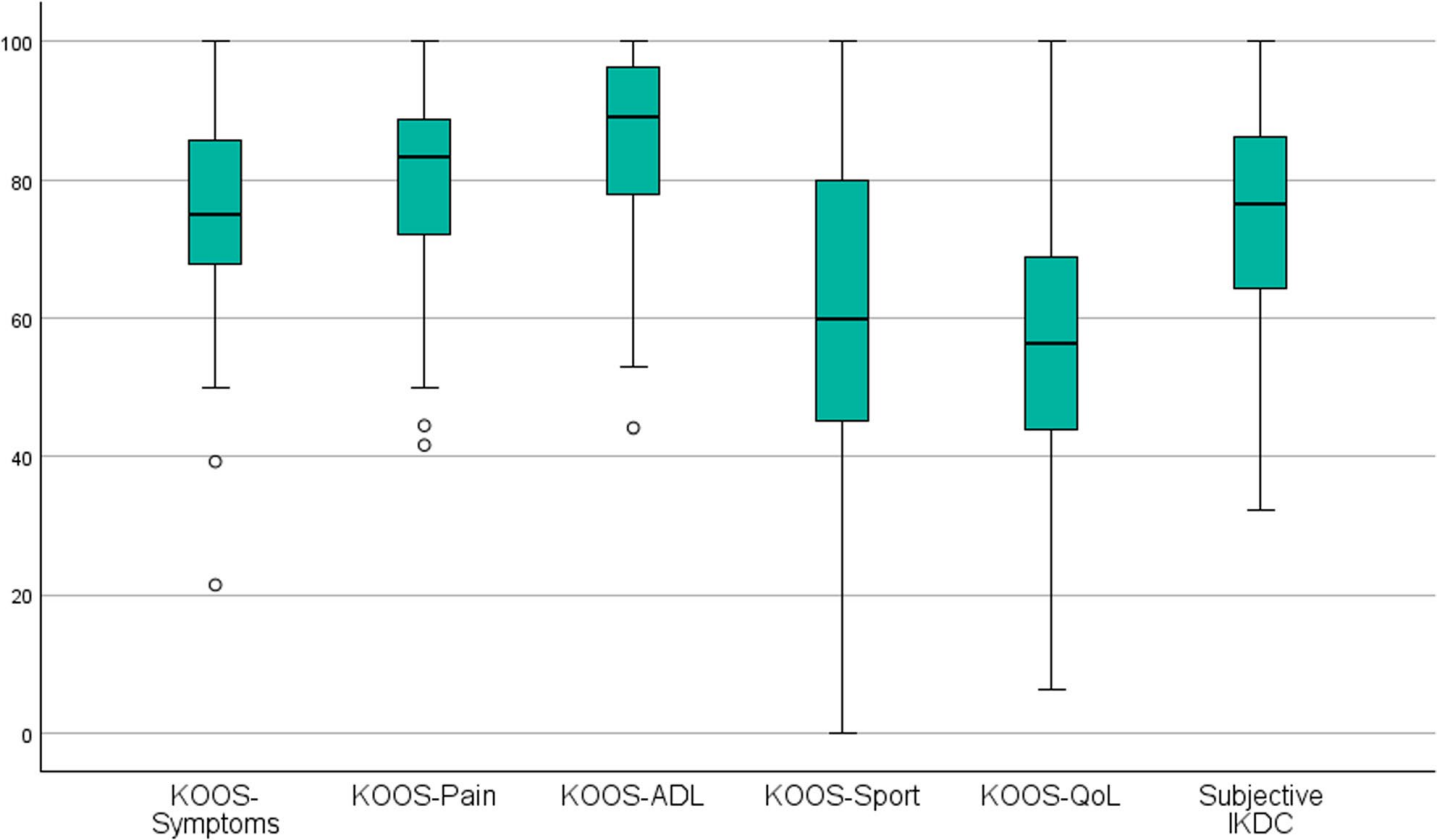
Boxplots depicting the distribution of the most recent KOOS subscale scores and the subjective IKDC score across all patients. Circles indicate statistical outliers

The mean interval between the most recent KOOS assessment and the clinical follow-up examination was 28.7 ± 19.4 months (range 3–85). The mean subjective IKDC score at follow-up was 74.6 ± 15.6. Mean KOOS subscale scores were 79.1 ± 14.6 for Pain, 75.5 ± 15.7 for Symptoms, 85.7 ± 13.1 for Activities of Daily Living, 59.2 ± 26.1 for Sport, and 55.5 ± 19.1 for Quality of Life. Higher scores indicate better subjective perception of knee health. The distribution of PROM scores is illustrated in (Fig. 1). Detailed results are summarized in (Table 2). The number of available observations varied between KOOS subscales due to missing registry data.

**Table 2:**
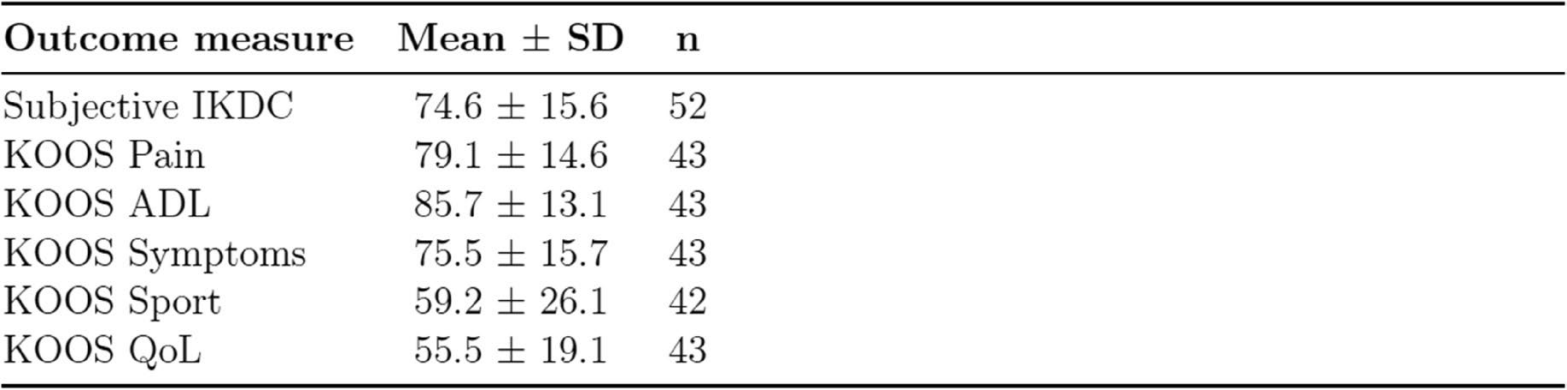
PROM scores

Values are presented as mean ± standard deviation (SD). Higher scores indicate better subjective perception of knee health. The number of available observations differed between KOOS subscales due to missing registry data

*Abbreviations*: *PROMs* Patient-reported outcome measures, *KOOS* Knee injury and Osteoarthritis Outcome Score, *IKDC* International Knee Documentation Committee, *ADL* Activities of Daily Living, *QoL* Quality of Life

### Functional performance test results

Functional performance testing revealed largely preserved global functional capacity while dynamic unilateral tasks demonstrated relevant side-to-side asymmetries. Detailed results are summarized in (Table 3).

**Table 3:**
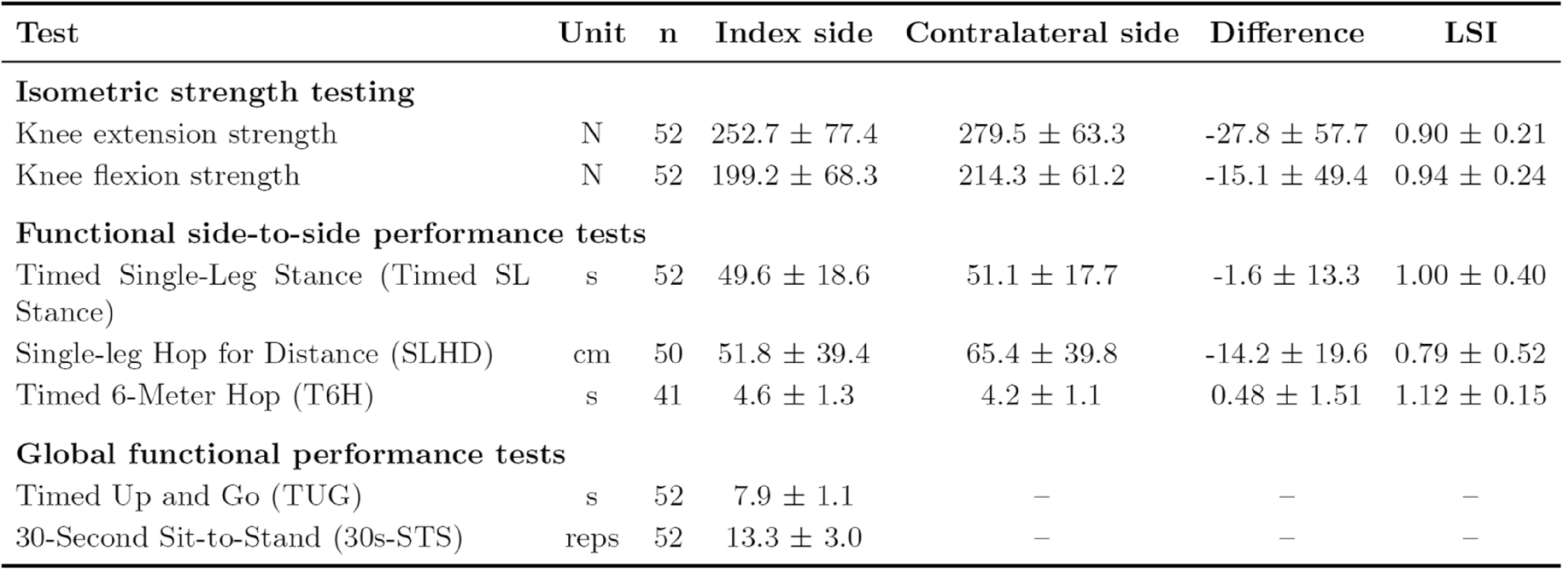
Strength and functional performance outcomes

Values are presented as mean ± standard deviation. Difference denotes Index side minus Contralateral side. LSI, Limb Symmetry Index, was calculated as Index side divided by Contralateral side (Index/Contralateral). LSI values < 1 indicate reduced performance of the index limb relative to the contralateral side, whereas values > 1 indicate superior performance

*SLHD* Single-leg Hop for Distance, *T6H* Timed 6-Meter Hop, *TUG* Timed Up and Go, *30s*-*STS* 30-Second Sit-to-Stand, *SL*, Single-Leg

The TUG was completed by all patients with a mean time of 7.9 ± 1.1 s. In the 30s-STS test patients completed a mean of 13.3 ± 3.0 repetitions. Performance in the Timed Single Leg Stance test was largely symmetric between limbs (LSI 1.0 ± 0.4). A ceiling effect was observed, with more than 70% of patients reaching the maximum standing time of 60 s on both sides, limiting the sensitivity of this test to detect subtle balance deficits.

In contrast, the SLHD revealed relevant asymmetries in dynamic performance. Patients showed reduced hop distance on the index limb compared to the contralateral side, with a mean LSI of 0.79 ± 0.52 and a mean absolute difference of -14.2 ± 19.6 cm. Two out of 52 patients were not able to perform a single leg hop and were therefore excluded from this analysis. The T6H test could not be successfully completed by all patients on both limbs. To avoid misleading numerical values in cases of test failure, no artificial times were assigned. Instead, patients were categorized according to their LSI with higher categories indicating poorer functional performance of the index limb. Patients who were unable to complete the task with their index limb were then entered in the highest category. Among patients able to complete the test on both limbs, the mean T6H time was 4.6 ± 1.3 s on the index limb and 4.2 ± 1.1 s on the contralateral limb (Table 3). The distribution of patients across performance categories, including the predefined category definitions, is shown in (Table 4).

**Table 4:**
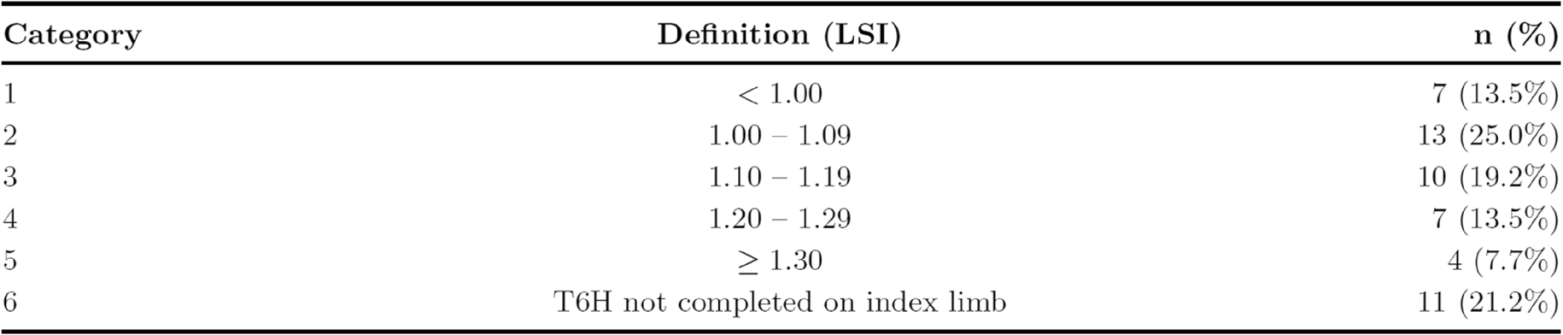
Distribution of patients according to functional performance categories in the T6H

Categories are based on the Limb Symmetry Index (LSI) of the Timed 6-Meter Hop (T6H), calculated as Index limb divided by Contralateral limb (Index/Contralateral). Higher category levels indicate poorer functional performance of the index limb relative to the contralateral side. Category 6 represents patients unable to complete the T6H with the index limb

*Abbreviations*: *LSI* Limb Symmetry Index, *T6H* Timed 6-Meter Hop

Isometric knee extension and flexion strength were assessed bilaterally and expressed as LSI. Overall, patients demonstrated reduced strength on the index limb compared to the contralateral side. Strength asymmetries were more pronounced for knee extension (mean LSI 0.90 ± 0.21) than for knee flexion (mean LSI 0.94 ± 0.24). Detailed strength values are presented in (Table 3).

### Correlations between PROMs and functional performance

Correlations between isometric strength symmetry and PROMs differed between knee flexion and extension. Flexion strength symmetry showed only weak and non-significant associations with all PROMs (all ρ < 0.25, all *p* > 0.17). In contrast, extension strength symmetry demonstrated a moderate positive correlation with the subjective IKDC score (ρ = 0.416, *p* = 0.002) and with the KOOS Sport subscale (ρ = 0.363, *p* = 0.020), whereas correlations with the remaining KOOS subscales did not reach statistical significance (Table 5).

**Table 5:**
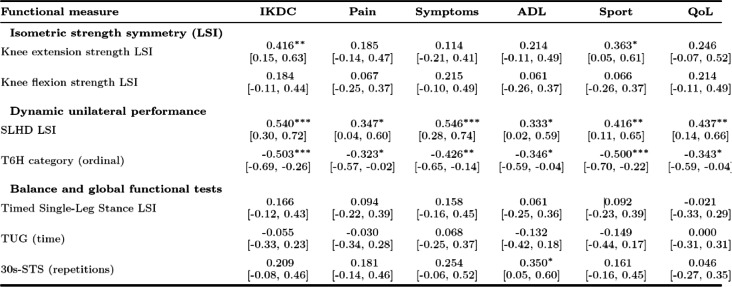
Spearman correlations between PROMs and functional performance measures

Values are Spearman's rank correlation coefficients (p) [95% confidence intervals]. Limb Symmetry Index (LSI) was calculated as Index limb divided by Contralateral limb (Index/Contralateral). The T6H category was treated as an ordinal variable; higher category levels indicate poorer functional performance of the index limb relative to the contralateral side, which explains negative correlations with PROMs (higher PROM scores indicate better perceived knee health)

*Abbreviations*: *PROMs* Patient-reported outcome measures, *IKDC* International Knee Documentation Committee (subjective IKDC score), *KOOS* Knee injury and Osteoarthritis Outcome Score, *ADL* Activities of Daily Living, *QoL* Quality of Life, *SLHD* Single-leg Hop for Distance, *T6H* Timed 6-Meter Hop, *TUG* Timed Up and Go, *30s*-*STS* 30-Second Sit-to-Stand

*p < 0.05, ** p < 0.01, *** p < 0.001. Pain, Symptoms, ADL, Sport, and QoL refer to the respective KOOS subscales. 30-Second Sit-to-Stand

The SLHD LSI showed the strongest and most consistent associations with PROMs among all functional tests. Reduced hop symmetry was significantly associated with worse subjective knee function across all PROM domains. Strong correlations were observed with the subjective IKDC score (ρ = 0.540, *p* < 0.001) and KOOS Symptoms (ρ = 0.546, *p* < 0.001). Moderate correlations were found with KOOS Pain (ρ = 0.347, *p* = 0.026), KOOS ADL (ρ = 0.333, *p* = 0.033), KOOS Sport (ρ = 0.416, *p* = 0.008) and KOOS QoL (ρ = 0.437, *P* = 0.004) (Table 5).

For the T6H test, performance asymmetry also showed significant associations with PROMs. The T6H category as previously explained demonstrated a strong negative correlation with the subjective IKDC score (ρ = -0.503, *p* < 0.001) and with the KOOS Sport subscale (ρ = -0.500, *p* < 0.001). Moderate negative correlations were observed with KOOS Symptoms (ρ = -0.426, *p* = 0.004), KOOS ADL (ρ = -0.346, *p* = 0.023), KOOS Pain (ρ = -0.323, *p* = 0.035), and KOOS QoL (ρ = -0.343, *p* = 0.025) (Table 5). Negative correlation coefficients reflect the reverse scaling of the T6H categories where higher category levels represent worse functional symmetry, whereas higher PROM scores indicate greater subjective knee function.

No significant associations were observed for the Timed Single Leg Stance test (all *p* > 0.2) or the TUG test (all *p* > 0.3) and any PROM (Table 5). Notably, a pronounced ceiling effect was observed for the Timed Single Leg Stance test with 71.2% of patients reaching the maximum predefined testing duration of 60 s on the index limb and 76.9% on the contralateral limb. For the 30s-STS test, a single moderate association with the KOOS ADL was observed (ρ = 0.350, *p* = 0.021). All other recorded PROMs showed no statistically significant correlation (all *p* > 0.1) (Table 5).

### Sensitivity analysis

To evaluate the potential impact of the temporal discrepancy between KOOS assessments and functional testing, a sensitivity analysis was performed by restricting the analysis to patients with an interval of 36 months or less (*n* = 29). This temporal adjustment resulted in a strengthening of some of the primary subjective-objective associations. Specifically, for the SLHD LSI, the correlation with KOOS Symptoms increased from the primary analysis to a highly significant ρ = 0.685 (*p* < 0.001), while significant associations were maintained for KOOS Sport (ρ = 0.394, *p* < 0.035) and KOOS QoL (ρ = 0.375, *p* = 0.045). Due to the reduced sample size, the associations for KOOS Pain (ρ = 0.302, *p* = 0.111) and KOOS ADL (ρ = 0.325, *p* = 0.086) did not reach statistical significance but remained stable in magnitude compared to the initial analysis.

For the T6H category, the correlations showed similar trends. KOOS Symptoms showed a strong negative correlation of ρ = -0.625 (*p* < 0.001) and KOOS Sport remained highly significant at ρ = -0.518 (*p* = 0.004). Due to reduced sample size, the moderate associations for KOOS Pain (ρ = -0.358, *p* = 0.056) and KOOS ADL (ρ = -0.375, *p* = 0.057) and KOOS QoL (ρ = -0.272, *p* = 0.152) did not reach formal statistical significance but closely approached the significance threshold.

## Discussion

The present study aimed to explore the relationship between subjective PROMs and objective functional performance tests in patients after cartilage repair surgery of the knee. The results show test-dependent differences in the degree of correlation with PROMs. Specifically for isometric extension strength and single-leg hop tests, moderate to strong correlations were demonstrated. Static and bilateral functional tests and isometric flexion strength did not correlate consistently with the subjective IKDC score and KOOS subscales. The subjective IKDC score was associated most strongly and consistently with the functional tests among all investigated PROMs.

Unilateral dynamic jump tests capture multiple dimensions of knee function but also coordination, trust in the index knee and movement safety at the same time. Their association with PROMs was more direct and reliable than those of less complex tests such as the Timed Single Leg Stance, TUG or 30s-STS. This indicates that more complex tests might be better aligned with subjective function in this specific patient cohort than bilateral and less demanding tasks.

The subjective IKDC score showed the most consistent, strongest correlations for multiple performance tests. The score aims to capture multiple domains of knee health within a single score rather than being separated in subscales like the KOOS. As complex functional tests also capture multiple domains of knee health and performance, this conceptual alignment between measurement instruments might be part of the reason for those correlations. Yet PROMs still differ from objective performance measures in their score and interpretation. This has been highlighted previously in findings in patients after anterior cruciate ligament reconstruction. Logerstedt et al. demonstrated that low subjective IKDC scores were strongly associated with failure to meet objective return-to-activity criteria, whereas normal IKDC scores did not reliably predict successful performance in functional test batteries [14]. Taken together, these results indicate that the subjective IKDC score is effective at identifying relevant functional impairments but cannot substitute objective performance testing when functional readiness needs to be confirmed. It indicates that the subjective IKDC score reflects objective knee performance more directly than the fragmented view of each KOOS subscale on its own.

The TUG and Single Leg Balance Test did not show any statistically significant correlation with any PROM. For the Single Leg Balance Test this might be due to the observed ceiling effect, with more than 70% of patients achieving the cut-off at 60s balance time. The observed patient cohort was relatively young, scored high in PROMs and showed overall good LSI. The Single Leg Balance Test was not sensitive enough to detect subtle balance deficits in this cohort. A similar pattern is true for the TUG. During the follow-up examination the time for completing the distance seemed to depend more on the patient’s interpretation of a normal walking pace, rather than their physical capabilities. It is important to note that the lack of correlation does not equal lack of clinical relevance, but rather a limited capability of those low-demand tests to detect functional deficits in this specific cohort. These results should therefore be interpreted with caution concerning older or functionally more restricted cohorts.

Discrepancies between PROMs and objective data have been shown in previous studies. In a study by Ekanayake et al. (2022) patients with end-stage osteoarthritis of the knee the KOOS Joint Replacement (KOOS-JR) only showed weak to no correlations with a functional assessment, using a markerless movement analysis [15]. Also, no statistically significant correlations could be demonstrated in a study by Oettl et al. (2025). Here, changes in PROMs (subjective IKDC score and COMI-knee score) were correlated with the objective MOCART and MOCART 2.0 scores which are based on MRI-findings in the knee joint [7]. The results of the present study, which show only moderate correlations between subjective PROMs and objective findings, are therefore consistent with the existing literature and expand the results to a patient cohort after knee cartilage repair surgery. The results are also consistent with previous findings from our group, which demonstrated only moderate associations between PROMs and an objective clinical knee score after cartilage repair surgery [20].

PROMs capture subjective aspects of knee health and are a great tool to measure the development of knee symptoms and patient satisfaction. On the contrary, functional performance tests are essential to measure objective knee performance. A key message of our findings is therefore, that PROMs play a critical role in capturing a complete image of knee health, yet cannot replace objective functional performance tests as they only show moderate correlations with some of the tests. Rather a combination of subjective PROMs and objective functional and clinical parameters is needed to fully capture multiple dimensions of knee health. This is of high relevance specifically in return-to-sports decisions and the management of rehabilitation.

As the patient cohort in this study was limited in size, further studies with larger cohorts should aim to reproduce the results presented in this study. As differences in correlation with objective findings between the KOOS subscales and subjective IKDC score were demonstrated, further research should focus on evaluating which specific PROM is suitable to detect which aspect of knee health and functional performance. This would be both important for clinical decision making, but also interpretation of medical registry data, as often PROMs are used as primary endpoints in registries.

A limitation of this study was the temporal discrepancy between the KOOS assessments and the follow-up examination, with a mean interval of nearly 29 months. To evaluate whether this interval introduced a systematic bias, a sensitivity analysis was performed by restricting the cohort to patients with a follow-up interval of 36 months or less (*n* = 29). While the reduced sample size in the sensitivity analysis led to a loss of formal statistical significance for some KOOS-subscales, the consistency of the correlation coefficients and the clear trends observed confirm the robustness of the primary findings.

Further limitations should be considered when interpreting the results of the study. First, the monocentric design and relatively small sample size limit generalizability and statistical power. In addition, participation in the follow-up examination was voluntary, introducing a potential selection bias towards patients with better functional outcomes. Second, follow-up examinations were not conducted at standardized time interval after surgery. This, together with the temporal latency between registry-based KOOS assessments and the follow-up examination, might have weakened observed correlations, particularly for the KOOS. Third, ceiling effects were observed for the Single Leg Stand test and the TUG appeared insufficiently sensitive to detect deficits in a relatively young and functionally competent cohort. Consequently, the informative value of those tests may be limited in this population. Fourth, the investigator was not blinded to the patients’ clinical status, which introduces a potential source of detection bias. However, standardized testing instructions and objective measurement tools (i.e., a handheld dynamometer and digital stopwatch) were strictly utilized. Another limitation is the heterogeneity of the study cohort regarding the cartilage repair procedures performed. Different surgical techniques are associated with distinct indications, rehabilitation protocols, and long-term clinical outcomes. Consequently, both PROMs and objective functional performance may have been influenced by procedure-specific factors. Because the primary objective of this study was to explore the association between subjective and objective outcome measures across a real-world cartilage repair population, all procedures were analyzed together. However, it cannot be excluded that procedure-specific differences affected the observed correlations. The use of limb symmetry indices (LSIs) as the primary measure of unilateral functional performance can also be partly problematic. LSIs assume that the contralateral limb represents an appropriate reference for normal function. However, unrecognized pathology of the contralateral knee or bilateral deconditioning may influence side-to-side comparisons and potentially result in an overestimation of functional recovery. Lastly, this study was exploratory in nature, conducted without an a-priori power calculation, and no statistical corrections for multiple testing were applied. This approach increases the risk of a Type I error, meaning that certain correlations may represent false-positive findings. In particular, associations with borderline significance such as some correlations between specific KOOS subscales and functional scores approaching the 0.05 threshold must be interpreted with caution. These individual findings require validation in larger, confirmatory cohorts. Therefore, the overall results should be interpreted as hypothesis-generating.

## Conclusion

In patients after knee cartilage repair surgery, patient-reported outcome measures showed only moderate and test-dependent associations with objective functional performance. The strongest correlations were observed for dynamic unilateral tasks and knee extension strength, while low-demand and bilateral tests were not sufficiently sensitive to detect functional deficits in this relatively high-functioning cohort. The subjective IKDC score demonstrated the most consistent relationships with performance-based measures. These findings indicate that PROMs capture relevant aspects of perceived knee function but cannot replace objective functional testing. A combined assessment integrating subjective and objective outcome measures is therefore essential for comprehensive evaluation of knee function and informed rehabilitation and return-to-sport decision making.

## Data Availability

The datasets generated and/or analyzed during the current study are available from the corresponding author on reasonable request.

## Abbreviations

ACT: Autologous Chondrocyte Transplantation
ADL: Activities of Daily Living
BMI: Body Mass Index
COMI: Core Outcome Measures Index
IKDC: International Knee Documentation Committee
KOOS: Knee Injury and Osteoarthritis Outcome Score
KOOS–JR: Knee Injury and Osteoarthritis Outcome Score for Joint Replacement
LSI: Limb Symmetry Index
MOCART: Magnetic Resonance Observation of Cartilage Repair Tissue
MRI: Magnetic Resonance Imaging
OCT: Osteochondral Transplantation
PROM: Patient–Reported Outcome Measure
QoL: Quality of Life
SLHD: Single–Leg Hop for Distance
SPSS: Statistical Package for the Social Sciences
STS: Sit–to–Stand
T6H: Timed 6–Meter Hop
TUG: Timed Up and Go
